# Neuroinflammation in epileptogenesis: from pathophysiology to therapeutic strategies

**DOI:** 10.3389/fimmu.2023.1269241

**Published:** 2023-12-22

**Authors:** Wenjun Li, Jinze Wu, Yini Zeng, Wen Zheng

**Affiliations:** Department of Neurology, Third Xiangya Hospital, Central South University, Changsha, China

**Keywords:** epileptogenesis, seizures, neuroinflammation, microglia, astrocytes

## Abstract

Epilepsy is a group of enduring neurological disorder characterized by spontaneous and recurrent seizures with heterogeneous etiology, clinical expression, severity, and prognosis. Growing body of research investigates that epileptic seizures are originated from neuronal synchronized and excessive electrical activity. However, the underlying molecular mechanisms of epileptogenesis have not yet been fully elucidated and 30% of epileptic patients still are resistant to the currently available pharmacological treatments with recurrent seizures throughout life. Over the past two decades years accumulated evidences provide strong support to the hypothesis that neuroinflammation, including microglia and astrocytes activation, a cascade of inflammatory mediator releasing, and peripheral immune cells infiltration from blood into brain, is associated with epileptogenesis. Meanwhile, an increasing body of preclinical researches reveal that the anti-inflammatory therapeutics targeting crucial inflammatory components are effective and promising in the treatment of epilepsy. The aim of the present study is to highlight the current understanding of the potential neuroinflammatory mechanisms in epileptogenesis and the potential therapeutic targets against epileptic seizures.

## 1 Introduction

Epilepsy is a variety of enduring neurological disorder characterized by tendency to spontaneous and recurrent seizures with heterogeneous etiology, pathogenesis, clinical features, and outcomes (1, 2). This disorder is serious global health issue affecting over 50-70 million people in the world (3). Even though a huge number of clinical and basic epileptic researches have been done, approximately 60% of epileptic disorders have unknown pathophysiological basis responsible for the occurrence and recurrence of seizures and 30% of epileptic patients still are resistant to the current pharmacotherapies with recurrent seizures throughout life (4, 5). As a result of these facts, it requires a more thorough understanding of the pathophysiological mechanisms in epileptogenesis, as well as exploring more effective therapeutic strategies for epileptic patients.

Growing body of researches investigate that epileptic seizures are originated from neuronal synchronized and excessive electrical activity, which may be a result of imbalance between glutamatergic and gamma-aminobutyric acid (GABA)ergic signaling induced by pathological factors including aberrant ion channel activation, neurotransmitter release, immune cells activation, etc. (4, 6, 7). Over the past two decades years growing evidences from both clinical and basic studies provide strong support to the conclusion that neuroinflammation is involved in epileptogenesis or a lot of neurological diseases with recurrent epileptic seizures (2, 4, 6).

Neuroinflammation, triggered by a number of exogenous or endogenous conditions including ageing process, gene expression variation, cerebral homeostasis disruption, pathogen infections, autoimmune diseases, brain tumor, stroke, and brain trauma, etc. (1, 4, 8–10), involves in a cascade of innate and adaptive immune responses characterized by the activation of microglia, astrocytes and endothelial cells (9), peripheral recruitment of multiple immune cells including but not limited to leukocytes, monocytes and lymphocytes into brain, and releasing a wide variety of inflammatory molecules, such as cytokines, chemokines, and growth factors, etc. (1, 4, 6, 8–10). Accumulated evidences show neuroinflammation is involved in neuronal hyperexcitability and epileptic seizures, as well prolonged epileptic seizures can trigger a chain of neuroinflammatory reactions (4, 11, 12). Neuroinflammation and recurrent seizures can be the mutual initiation factors and consequent reactions, which collectively contribute to devastating clinical consequences (5, 13). Meanwhile, an increasing body of preclinical researches reveal that the anti-inflammatory therapies specifically targeted a certain inflammatory component are effective and promising in animal models with seizures and epilepsy (14–16).

The aim of the present study is to highlight the update understanding of the underlying inflammatory mechanisms in diverse epileptic seizures, with particular emphasis on the neuroinflammatory pathways in epileptogenesis and potential therapeutic targets in epilepsy.

## 2 Activated microglia in epilepsy

**Figure 1:**
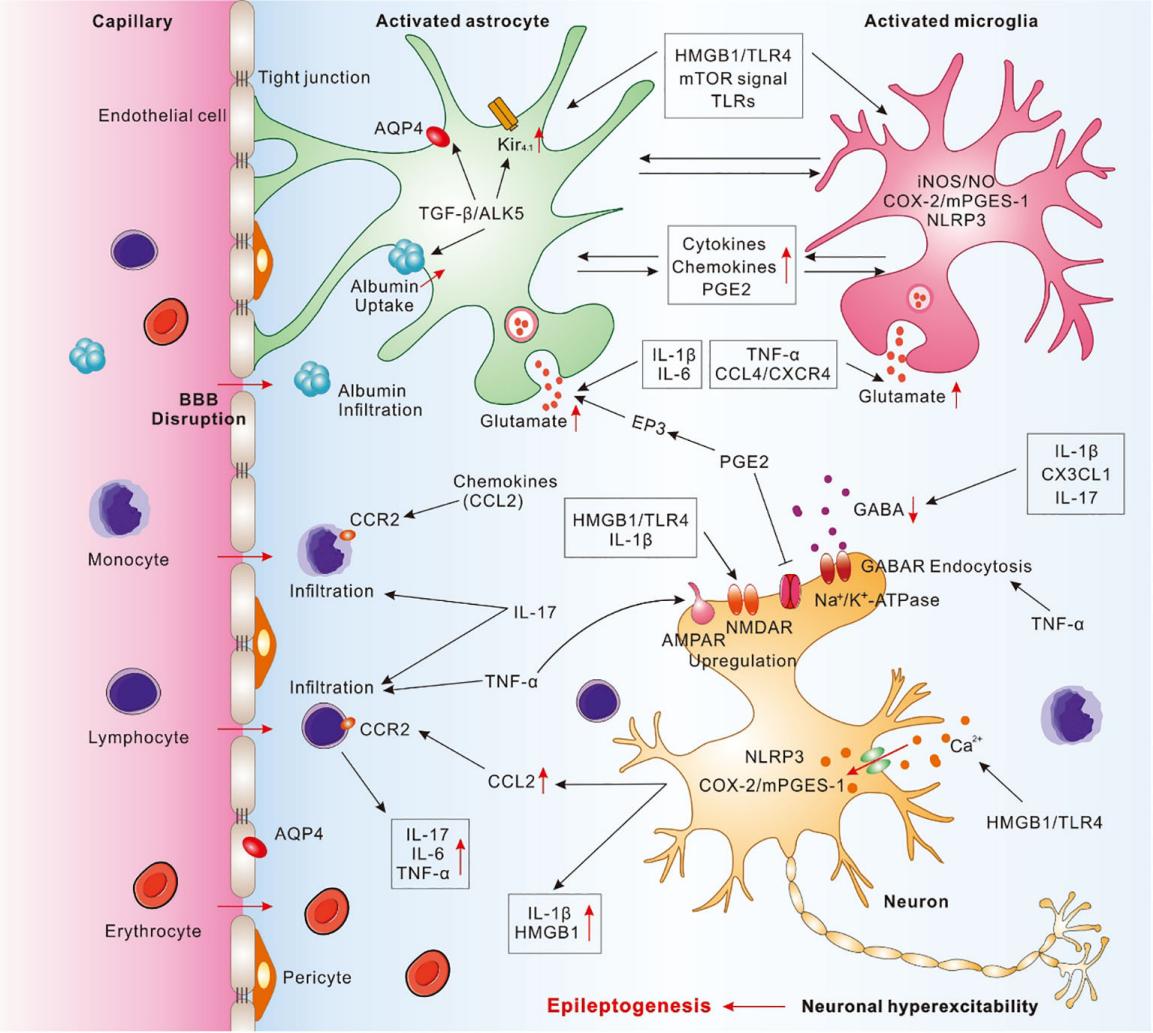
Pathophysiological cascade of inflammatory mechanisms in epilepsy. Activated astrocytes and microglia may cooperate with each other and release a wide range of pro-inflammatory molecules to mediate neuroinflammation, contributed to the neuronal hyperexcitability and epileptogenesis. IL-1β, secreted by activated microglia, astrocytes and neurons, may enhance glutamate release, decrease glutamate reuptake, and decrease GABA_A_ currents. IL-6, secreted from activated astrocytes and microglia, can promote glutamate release. IL-17, secreted from astrocytes, microglia, and T-lymphocytes, may promote the infiltration of peripheral immune cells, inhibit GABA-induced inhibitory synaptic transmission, and result in excitation-inhibition imbalance. TNF-α, secreted by activated microglia and astrocytes, may promote infiltration of peripheral T lymphocytes, trigger microglial glutamate release, and induce GABA receptor endocytosis. HMGB1, released by activated astrocytes, microglia, or neurons, may interact with TLR4 and promote the pro-inflammatory cytokines release, increase Ca^2+^ influx, upregulate NMDAR, and destruct BBB. Activation of TGF-β/ALK5 signaling in astrocytes may mediate albumin uptake after BBB disruption, downregulate Kir_4.1_ channel, and impair AQP4. Chemokines, including CCL2, CCL3, CCL4, CX3CL1, and CXCL13, etc., may be secreted from astrocytes, microglia, and endothelial cells and take a crucial role in immune cells migrating from blood to brain through binding to corresponding receptor, such as CCL2-CCR2. PGE2, mainly secreted from activated astrocytes and microglia induced by COX-2/mPGES-1 axis in microglia, neurons, and endothelial cells, may inhibit the neuronal Na^+^/K^+^-ATPase. mTOR signal may activate astrocytes and microglia, promote the release of pro-inflammatory cytokines and chemokines, as well as disrupt BBB. TLRs, expressed by microglia and astrocytes, may trigger cerebral innate immune response and promote the release of pro-inflammatory cytokines. NLRP3 inflammasome elevated in neurons and microglia may activate Caspase-1 and increase the release of pro-inflammatory cytokines.

Microglia, usually known as cerebral macrophages, may activate distinct physiological action in brain following the polarization of pro-inflammatory M1 cell subtype or anti-inflammatory M2 cell subtype, despite the underlying mechanisms and cell interactions between two phenotypes regulating are largely unknown (17–19). The pathophysiological consequences of microglia activation include aggravating inflammation, regulating neuronal activity, endocytosing dead neurons, and inducing epileptic seizures (7, 20–22). Growing evidences show that activated microglia may downregulate the convulsive threshold in experimentally-induced epileptic models (2, 17, 23, 24). The prolonged or excessive microglial activation may secret and release a wide range of inflammatory cytokines and chemokines, which is obviously observed in epileptogenesis (**Figure 1**) (11, 18, 25). The expression level of these inflammatory molecules is associated with the seizure severity in animal models or epileptic patients (17, 26). In addition, activated microglia may be in cooperation with astrocytes and in turn promote astrocytic glutamate release, which contribute to seizures and cell loss (2, 17).

Activated microglia may generate high levels of nitric oxide (NO) via inducing inducible nitric oxide synthase (iNOS). Subsequently NO activates nicotinamide adenine dinucleotide (NADPH) oxidase and promotes the releasing of pro-inflammatory mediators such as tumor necrosis factor-α (TNF-α) (4). Moreover, the releasing of pro-inflammatory mediators and iNOS may be regulated by intrinsic astrocytic signals, such as the mitogen-activated protein kinase (MAPK) and the nuclear factor-кB (NF-кB) axis (27). Accumulating evidences demonstrate that the expression of NO, iNOS, NF-кB, and MAPK are significantly increased in a variety of epileptic models (4, 28).

Moreover, it has been substantiated that non-inflammatory action mediated by microglia is thus adequate to generate epileptic seizures (22, 29). Several studies report that microglia are involved in the neurogenesis in the epileptic brain. In animal models with KA-induced SE, microglia suppress neurogenesis by Toll-Like Receptor 9 (TLR9)-induced TNF-α secretion and decreasing ectopic granule cells (30). Through suppressing the excessive proliferating of neural stem cells (NSCs) and eliminating adult-born granule cells (GCs), activated microglia restrain the generation of abnormal brain circuits after SE, which may disrupt the balance between excitatory synapses and inhibitory synapses (21, 30). However, a body of other research finds that suppression of microglial activation can inhibit the neurogenesis after SE by decreasing the ectopic granule cells or immature neurons in different animal models (30, 31). These inconsistent results of microglia mediating neurogenesis may be attribute to the different chemically induced animal models and the different pathophysiological conditions. Different gene expression of microglia is observed in several animal models including KA-induced and pilocarpine-induced animal models (20).

## 3 Activated astrocytes in epilepsy

Reactive astrocytes activation and astrogliosis, including the morphologic alteration, molecular expression, and proliferation, were observed in various cerebral regions of animal models or patients with epileptic seizures (17, 30, 32). By autocrine and paracrine actions, activated astrocytes release pro-inflammatory molecules, including IL-1β, high mobility group box 1 (HMGB1), nuclear factor Kappa-beta (NF-kB), etc., all of which may promote seizure onset and recurrence (17, 23, 25). IL-1β may couple with interleukin 1 receptor type 1 (IL1R1) and activate nuclear factors κB (NF-κB), which strongly contribute to seizure generation and progression (6, 28, 33). HMGB1 may activate IL-1R/Toll-like receptor 4 (TLR4) signals and stimulate NF-κB, which take a crucial role in seizure occurrence and duration (6, 34–37). It is reported that overactivations of astrocytes disturb the synaptic function and lead to neuronal apoptosis (17, 18). On the contrary, astrocytes can be activated by seizures and subsequently mediate neuroinflammation (38).

Apart from the releasing of pro-inflammatory mediators, astrocytes may change neuronal excitability and lead to pathogenesis of epilepsy by regulating the water and K^+^ flow, which is also involved in the epileptogenesis (17, 39). Aquaporin-4 (AQP4) is a protein expressed in astrocytes and endothelial cells, which take a crucial role in modulating interstitial fluid osmolarity (ISF) and extracellular fluid (ECF) volume. By upregulating the astroglial potassium channel (Kir4.1) expression and AQP4 function, activated astrocytes may result in neuronal hyperexcitability (30). In KA-induced SE model, AQP4 is markedly downregulated, and the homeostatic conditions of water and potassium may be broken in early stage of epileptogenesis (40). AQP4 knockout (KO) mice or AQP4 anchoring the dystrophin-associated protein complex may enhance seizure susceptibility (39).

Several evidences from recent researches suggest that astrocytic enzyme adenosine kinase (ADK) increases in of epileptic animal models, which can lower the seizure threshold by reducing extracellular adenosine (41–43). In addition, growing evidences show that astrocytes regulate the excitatory/inhibitory neurotransmitter homeostasis and control the neuronal activity by releasing, uptake, and storing various mediates (17, 41–43). Astrocytes take part in the removing most of extracellular glutamate via glutamate aspartate transporter (GLAST) and glutamate transporter-1 (GLT-1) (17). GLT-1 KO or selective inhibitor of GLT-1 enhance the concentration of synaptic glutamate and exhibit epileptic hyperexcitability and spontaneous lethal seizures (44, 45).

It is well known that astrocytes regulate neurogenesis, as well glutamate and GABA directly control neurogenesis. The level of nestin in astrocytes is upregulated in animal models with KA-induced severe seizures, which may promote the neuronal migration and location (46). The disruption of homeostasis between glutamate and GABA following astrocyte activation may trigger abnormal neurogenesis (47).

## 4 Peripheral immune cells in epilepsy

Accumulated evidences from recent researches strongly show that peripheral innate and adaptive immune cells take a major role in the pathogenesis of seizures and epileptogenesis (**Figure 1**) (2).

Blood monocytes can migrate into the brain through impaired blood-brain barrier (BBB) and subsequently mediate neuroinflammation by differentiating to macrophages or microglia-like cells, which is considered to involve in a wide range of cerebral disorders, like stroke, amyotrophic lateral sclerosis, and epilepsy (18, 22, 48–50). Emerging evidences show that the infiltrating of monocyte amplify the pathological effects of chemically kindled SE (48, 49). The migrate of monocyte depend on the chemokine (C-C motif) ligand 2 (CCL2)/C-C motif chemokine receptor 2 (CCR2) axis (48). CCL2 is extremely upregulated in activated microglia and neurons after SE in animal models or epileptic patients (48, 51). Blocking the CCL2/CCR2 signal pathway may alleviate the seizure occurrence in mice with mesial temporal lobe epilepsy induced by lipopolysaccharide (51). The infiltrating of monocyte may also contribute to the significant microglia activation and microgliosis after SE (50). The activated microglia is the predominant secreting cell of CCL2, and CCL2 subsequently promotes the monocyte recruiting by activating CCR2 (52). Infiltrating monocytes are gradually considered as novel promising therapeutic directions of seizures and epilepsy (18, 22).

As well as monocyte, lymphocytes are recruited into brain in epileptic neuroinflammation and mediate the adaptive immune response (26, 53). It was observed that pro-inflammatory blood-borne CD4^+^ and CD8^+^ T cells are recruited into neocortex and hippocampus in seizure animals or patients with epilepsy (54). Pro-inflammatory γδ T cells are increased in epileptic lesions, and their numbers are correspondingly associated with clinical outcomes in pediatric epileptic patients (55). It is noteworthy that regulatory T cells (Tregs), a kind of lymphocyte involved in immune regulation by inhibiting inflammation, may increase significantly following cerebral inflammation, which may alleviate seizure severity (56). The mice lacked T and B cells by silencing recombination activating gene 1 (RAG1) show significant neurodegenerating and recurrent seizures after KA administration (57). It is also reported that the deficiency of both γδ T cell- and IL-17RA aggravates KA-kindled SE in mice, as same as Tregs depletion (55).

## 5 Pro-inflammatory molecules in epilepsy

Pro-inflammatory molecules, such as cytokines, chemokines, and growth factors, etc., modulate neuroinflammatory processes and mediate the occurrence and development of epileptic seizures (**Figure 1**) (16, 18, 33, 58). A growing body of study has demonstrated cytokines, including IL-1β, IL-6, IL-17, TNF-α, and transforming growth factor-beta1(TGF-β1), etc., are significantly upregulated in brain following epileptic seizures, primarily secreted by glial cells and neurons (18, 59–61). Such crucial cytokines and homologous receptors involve in various pro-inflammatory pathways and result in neuronal hyperexcitability (59).

### 5.1 IL-1β

As a typical extracellular inflammatory cytokine, IL-1β is secreted by innate immune cells, including monocyte and macrophage. But in central nervous system (CNS), IL-1β is predominantly secreted by activated microglia, astrocytes, neurons, and oligodendrocytes (6, 62). The level of IL-1β can be instantly elevated in response to serious pathological courses via cleaving pro-IL-1β, which subsequently combines with IL1R1 and activates NF-κB to mediate the inflammatory process (6, 14, 34, 62). Compelling evidence has shown that IL-1β upregulated in serum, cerebrospinal fluid (CSF) and multiple brain regions of patients with epilepsy or in epileptic animal models (63). During the acute course of SE or the chronic course of epileptic seizures, IL-1β is predominantly released by activated microglia or astrocytes, which may exacerbate the epileptic seizure and cause more serious consequences (33). It is also demonstrated that exogenous IL-1β significantly enhances seizure severity and duration in rodents with KA-induced seizures (64). On the contrary, downregulation of IL-1β in rat hippocampus delays seizure occurrence and reduces seizure severity (65).

A wider breadth of research documented that IL-1β may disturb the balance between excitatory transmission and inhibitory transmission by enhancing glutamate releasing and decreasing glutamate re-uptake (66), which may lead to neuronal hyperexcitability and excitotoxicity (6). IL-1β can enhance the glutamate transmission of excitatory neuron by activating of the N-methyl-D-aspartate (NMDA) receptor subunit 2B (GluN2B) (67). In addition, overexpression of IL-1β may decrease the GABA_A_ current (up to 30%) in temporal lobe epilepsy and rats with chronic epilepsy (68).

### 5.2 IL-6

As a pro-inflammatory cytokine, IL-6 takes a crucial modulating role in neuroinflammatory responses (69). IL-6 expresses in lower level in normal physiological conditions, but is significantly secreted from activated astrocytes and microglia, which may be regulated by different cytokines including TNF-α, IL-Iβ, and IL-17, etc. (70, 71). In epileptic patients and multitudinous seizure models, the expression of IL-6 obviously increases in various cerebral region like hippocampus, cortex, thalamus, and hypothalamus, as well as in blood (6). Different from other pro-inflammatory cytokines, the plasma level of IL-6 may increase significantly up to 24 h after seizures (72).

IL-6 can upregulate glutamate release, inhibit long-term potentiation (LTP), decrease hippocampal neurogenesis, and promote glosis, which significantly enhance neuronal excitability and mediate epileptogenesis (6, 70). A growing body of researches have demonstrated that IL-6 facilitate the seizure occurrence and exacerbation of epilepsy (73, 74). The mice overexpressed IL-6 in astrocytes is more subject to seizures after injection of KA (70, 73). Exogenous IL-6 may distinctly increase the severity of pentylenetetrazol (PTZ)-induced seizures (70, 74). The increasing of IL-6 and IL-1β in hippocampus during pregnancy may lead to neuronal hyperexcitability and development of seizures in offspring (75). However, the molecular mechanism underlying IL-6 contributing to seizures is not complete understood. Some other studies showed that genetic ablation or knockout of IL-6 activated oxidative stress-related signaling, increased neuronal damages, enhanced the susceptibility to seizures, and leaded to higher mortality rates in multiple chemoconvulsant models (76). Therefore, a large amount of research is required to identify the crucial roles and mechanisms of IL-6 in epileptogenesis and disease-modifying therapy.

### 5.3 IL-17

IL-17 is a large family of cytokines consisting of IL-17A, IL-17B, IL-17C, IL-17D, IL-17E, and IL-17F (77). The IL-17A and IL-17F cooperate to compose a dimer and subsequently bind to receptor complex IL-17RA/C, including IL-17 receptor A and IL-17 receptor C. The activating of IL-17 signal pathways may recruit adaptor molecule actin-related gene 1 (Act1) to the SEFIR domain of IL-17RA/C complex, subsequently recruit TNF receptor associated factor 6 (TRAF6) and activate a wide spectrum of pro-inflammatory signal pathways in brain (78).

Several previous studies found that IL-17 is primarily secreted from CD4^ +^ T cells, however, it is demonstrated that astrocytes are the predominant secreting cells of IL-17 in brain (79). Recent researches reveal that IL-17 is obviously increased in multiple innate and adaptive immune cells or nonimmune cells, such as dysmorphic neuron, balloon cell, giant cell, microglia, and T-lymphocyte, following the serious epileptic seizures (80). The elevated IL-17 in brain can activate microglia, promote the infiltrating of peripheral immune cells, and increase the secretion of pro-inflammatory mediators, including IL-6, IL-1β, and TNF-α, etc. (58, 81). At the same time, the upregulated pro-inflammatory mediators may inversely induce the releasing of IL-17 (82).

Accumulated evidences from recent studies support that IL-17 is closely related to epileptogenicity, and the IL-17 signal pathways may be an underlying antiepileptic treatment target (5). Increased IL-17 results in neuronal hyperexcitability in brain slice cultures (55). In variable types of epileptic patients, increased IL-17 in serum or CSF positively associated with seizure severity (53). A recent research observes that the numbers of IL-17-secreting γδ T cell locating in epileptogenic lesions are closely related to seizure severity (5). The deletion of IL-17R in KA-induce SE animal model decreases neuronal excitability and alleviates the seizure activity (55). IL-17 may result in an imbalance between excitatory neural circuit and inhibitory neural circuit and induce neuronal excessive discharge by regulating neurotransmitters releasing (83). Another study demonstrates that IL-17 inhibits GABA-mediated inhibitory postsynaptic electrical activity and leads to neuronal hyperexcitability (79).

### 5.4 TNF-α

TNF-α is a pro-inflammatory cytokine predominantly secreted by activated microglia and activated astrocytes reacting to inflammatory conditions (84). Overexpression of TNF-α is associated with seizures, ataxia, and paresis in mice, accompanied by inflammatory pathological alterations in brain, including T lymphocyte infiltrating, reactive astrogliosis and microgliosis, and focal demyelination (85). In rats with amygdala kindling or KA-inducing seizures, the expression TNF-α is elevated and persists for up to several days in hippocampus, amygdala and cortex, followed by extensive neuronal damage (61).

TNF-α signaling can provoke glutamate release from microglia by upregulating glutaminase and α-amino-3-hydroxy-5-methyl-4-isoxazolepropionic acid (AMPA) receptor (86). Upregulated AMPA receptors contribute to calcium uptake and neurotoxicity (24). In addition, TNF-α may decrease inhibitory receptors of postsynaptic membrane via inducing GABA receptor endocytosis (24, 87). Furthermore, it is reported TNF-α regulated adhesion molecule N-cadherin and mediated the generation and development of both excitatory synaptic networks and inhibitory synaptic networks (88). These multiple roles of TNF-α on the glutamate receptors and GABA receptors, as well as excitatory and inhibitory synaptic networks, synergistically lead to neuronal hyperexcitability and epileptogenesis (59).

Although the mounting evidences show that TNF-α takes an important role in epileptogenesis, it is also reported that recombinant TNF-α inhibits seizure occurrence induced by PTZ (89). The available researches demonstrate that two different tumor necrosis factor receptor (TNFR) subtypes, TNFR1 and TNFR2, mediate these dichotomous pro-convulsive and anti-convulsive function (87). Compared with TNFR2, TNFR1 has higher binding ability to TNF-α (90). The low expression TNF-α predominantly binds to TNFR1 and takes a pro-convulsive action, and the continually accumulation of TNF-α further activates TNFR2 and triggers an anti-convulsive mechanism in epileptic brain (6). In preclinical researches, certain ligands of TNF receptor have come to exploring as underlying treatment targets in epilepsy, while it is still under controversy whether the therapeutics against epilepsy targeting TNF-α signaling may generate the serious risks of infection and tumorigenesis (59).

### 5.5 Transforming growth factor β

TGF-β is a multifunctional cytokine regulating a wide range of downstream signaling through activating TGF-beta type II receptor (TβRII), TGF-beta type I receptor (TβR1), activin-like kinase 5 (ALK5), SMAD protein complexes, and multiple mitogen-activated protein kinase (MAPK), etc. (91). TGF-β mediates a series of biological activities such as inflammatory responses, cell proliferation, differentiation, apoptosis, and embryogenesis (91).

Recent mounting evidences show that TGF-β signaling pathways are intricated in epileptogenesis (92). In resected brain regions from epileptic patients and animal models, the increased expression of TβR1 is observed in cortex, amygdala, and hippocampus, particularly in the piriform cortex (61, 93). Activation of TGF-β signaling pathways in astrocytes can mediate albumin uptake, potassium (K^+^) buffering, glutamate metabolism, pro-inflammatory mediators releasing, and synaptogenesis, which collectively lead to neuronal hyperexcitability and epileptogenesis (94, 95). TGF-β/ALK5 signaling pathways activated by intracerebroventricular injection of albumin may induce excitatory synaptogenesis and spontaneous seizures in mice (96). As well, activation of TGF-β/ALK5 signaling pathways in transgenic *ALK5* ^CA^ mice regulates the late stages of adult hippocampal neurogenesis (96, 97). Both excitatory synaptogenesis and hippocampal neurogenesis may be involved in epileptogenesis. It is also demonstrated that TGF-β signaling pathways mediates astrocytes to uptake serum albumin through disrupted BBB, downregulates of potassium channel Kir_4.1_ in astrocytes, and reduces extracellular K^+^ buffering, which may induce NMDA receptor-induced neuronal hyperexcitability and seizure-like discharges (94). Another research found that activation of the TGF-β signaling pathways in hippocampus upregulates glutamatergic associated genes and downregulated GABAergic associated genes in reactive astrocytes, followed by glia-neuron communication and hippocampal-kindling epileptiform activity in rats (92). The recurrent seizures mediated by TGF-β signaling pathways can be abolished by homologous pharmacological inhibitor or antagonist, such as SJN2511, SB431542, and losartan (97), which suggests targeting TGF-β signaling pathways is a prospective therapeutic strategy for acquired epilepsy.

### 5.6 HMGB1

HMGB1, a ubiquitous chromatin-binding protein, contains two DNA-binding domains (box A and box B) in N-terminus and take part in regulating transcription, translation, and repair of DNA (37). As a pro-inflammatory domain, box B can bind to Toll-like receptors (TLRs) to initiate a neuroinflammatory response (98, 99). Oppositely, box A is a competitive anti-inflammatory response domain to alleviate the inflammatory activity (98).

HMGB1 may be a potential clinical biomarker for prediction, diagnosing, and prognosing of seizures or drug-resistant epilepsy (100–102). Numerous studies demonstrate that serum HMGB1 increases after seizures according to the severity of seizures and epileptic discharges (34, 35, 102). It is also reported that the levels of HMGB1 in CSF are upregulated in patients with autoimmune epilepsy, accompanied by other upregulated pro-inflammatory cytokines and chemokines (103). In mice model with KA-induced seizures, the high levels of HMGB1 are observed in hippocampal, and the antagonists of HMGB1 or anti-HMGB1 monoclonal antibodies are able to alleviate occurrence and severity of seizures (25, 101, 104).

A growing body of research demonstrates that HMGB1 is generated from activated astrocytes and microglia, or neuron, subsequently interacts with the primary receptor TLR4 to involve in the pathophysiology of epilepsy (2, 34). The levels of HMGB1 and TLR4 increase in cerebral regions of patients with mesial temporal lobe epilepsy, focal cortical dysplasia type II (FCD-II) with epilepsy, or drug-refractory epilepsy, correlating with the severity of epilepsy (35, 100). Binding to TLR4, extracellular HMGB1 can activate IL-1β/IL-1R and TLR4 signals, subsequently promote NF-κB transporting into nucleus and result in release of numerous pro-inflammatory cytokines, which may be involved in epileptogenesis (6, 35–37). It is also reported that HMGB1/TLR4 signaling pathway significantly increases the Ca^2+^ influx and activates the NMDA receptor by phosphorylating NR2B subunit (105), which usually contribute to neuronal hyperexcitability and epileptogenesis (106). In KA-induced animal models, HMGB1 downregulates the level of glutamate decarboxylase 67 (GAD67), glutamate dehydrogenase 1 (GLUD1), and glutamate dehydrogenase 2 (GLUD2), upregulates the levels of intracellular glutamate and major histocompatibility complex II (MHC II), which collectively contribute to increasing the neuronal excitability and inducing epileptic seizures (107). Another mechanism of extracellular HMGB1 contributing to epilepsy is based on the destruction of the BBB (108).

### 5.7 Chemokines

Chemokines are a large family of small proteins released by astrocytes, microglia, and endothelial cells (38), which is primarily stimulated by pro-inflammatory cytokines (109). Through acting on specific G protein‐coupled receptors (GPCRs), chemokines take a crucial role in infiltration of immune cells from blood to brain, as well as modulating electrophysiology via regulating neurotransmitter releasing and voltage-dependent channels or G-protein-gated channels (6, 110, 111). Several researches observed that chemokines, including CCL2, CCL3, CCL4, fractalkine/CX3C chemokine ligand 1 (CX3CL1), chemokine ligand 13 (CXCL13), and the corresponding receptor CCR2, CCR5, C-X-C chemokine receptor 4 (CXCR4), CXCR5, highly upregulated in hippocampus or other cerebral regions of patients and animal models with epilepsy (6, 59). In patients with intractable epilepsy, chemokine CCL2 significantly upregulates in neuron, astrocyte, microglia, neural progenitor cell, and microvascular endothelial cell (111). In KA-kindled SE, high-level CCL2 in hippocampus activates CCR2 and leads to microglia activation, monocyte infiltration, and neuron death by triggering transcription 3 (STAT3) and IL-1β signals (48). In TLE patients, the upregulation of CXCR4 on microglia and astrocytes induces microglia to release TNF-α and glutamate (110). In the pilocarpine-induced SE, knockout of CCR2 prevents the monocyte migration and decreases the secreting of pro-inflammatory cytokines (49). In addition, inhibition of CCL2/CCR2 signals significantly alleviate the severity of seizures in the KA-induce SE model (51). In pilocarpine SE models, exogenous CX3CL1 enhances SE-induced neuronal loss, which can be reversed by the antibodies of CX3CL1 or CX3CR1 (112). In KA-induce rat models, downregulation of CCR5 expression delays seizure onset and decreases seizure severity, neuron damage, and neuroinflammation (113). All of these accumulating evidences suggest that the chemokines and downstream signal pathways mediate the interaction between neuroinflammation and epileptogenesis (51, 113).

### 5.8 Cyclooxygenase-2, microsomal prostaglandin E synthase-1, prostaglandin E2 and PGE2 receptors

Cyclooxygenase (COX) is the crucial enzyme in biosynthesis of prostanoid, including prostaglandin D2 (PGD2), PGE2, prostaglandin F2 alpha (PGF2α), prostaglandin I2 (PGI2), and thromboxane A2 (TXA2) (14). COX-1 isoform, expressed widely in body, primarily take a key role in homeostatic prostaglandins (14). COX-2 isoform, a major pro-inflammatory factor, is usually expressed at very low levels in normal cerebral tissue, but which significantly increases following inflammatory conditions, fever, and seizures (114, 115). Prostaglandin E synthase (PGES), existing in three isoforms including mPGES-1, mPGES-2, and cytosolic PGES (cPGES), is a downstream enzyme of COX cascade and contribute to synthesis of PGE2 from prostaglandin H2 (PGH2) directly (116). mPGES-1 combines with COX-2 to co-induce the synthesizing of PGE2 and promote inflammatory responses (116, 117).

Both COX-2 and mPGES-1 are observed to be synchronously upregulated in activated microglia, neurons, and endothelial cells, in response to a wide range of neurological disorders, including seizures and strokes (118, 119). In mice with pilocarpine-induced SE, the expressions of COX-2 and mPGES-1 increase in the hippocampus in very synchronous time-courses, along with the elevating of PGE2 (115, 120). The selective inhibitors of both COX-2 and mPGES-1 may largely reduce brain inflammation, shorten seizure duration, and reduce the seizure incidence in epileptic animal models (12, 117). In mice models with chemoconvulsant seizures, the deficiency of mPGES-1 may decrease the levels of PGE2, which blocks the releasing of pro-inflammatory cytokines, attenuates occurrence of seizures and neuronal damage in brain (12). In addition, emerging evidences show that the upregulation of mPGES-1 in endothelial cell promotes astrocytic glutamate releasing and results in excitotoxicity (121). It is also reported by several studies that COX-2 inhibitors including NS-398, indomethacin, celecoxib and mPGES-1 inhibitor BI1029539 prevent P-glycoprotein upregulation after seizures (122, 123), which may be associated to the development of drug-resistance seizures (124).

PGE2 is induced by COX-2/mPGES-1 axis and mainly secreted from activated astrocytes and microglia, which couple with homologous receptors, including EP1, EP2, EP3, and EP4, and mediate neuroinflammation, neuronal hyperexcitability and excitotoxicity by infiltrating of immune cells and upregulating of many pro-inflammatory factors (125, 126). A growing body of researches have investigated the roles of PGE2 and its receptors in the development of seizures. By mediating of EP1, EP3 or EP4 receptor, PGE2 takes an inhibitory action on Na^+^/K^+^-ATPase to upregulate neuronal excitability (127). In addition, PGE2 binding to receptor EP3 promotes astrocytic glutamate release, which disrupts the balance between both glutamatergic signaling and GABAergic signaling (125). In multiple chemically kindled epileptic models, the antagonists of EP1, EP3 or EP4 decrease epileptic susceptibility and delays seizure induction by decreasing extracellular glutamate concentrations (125, 128–130).

Differing from the EP1, EP3 or EP4 receptors, EP2 receptor usually affords beneficial and positive roles in brain after cerebral injury (6). The activation of EP2 may block brain-derived neurotrophic factor (BDNF)/tyrosine kinase receptor B (TrkB) signal pathway and prevent from acquired epileptogenesis after pilocarpine administration in mice (120, 131). However, recently accumulated evidences strongly demonstrate that EP2 activating may induce the secondary toxicity and damage in animal models with neuroinflammation or neurodegeneration (126, 132–134). Meanwhile, EP2 antagonist decreases neuronal death, reduces mortality, attenuates BBB breakdown, alleviates gliosis in the hippocampus of SE animal models (6, 135–137). In addition, several other studies show some inconsistent findings in COX-2 inhibitors, including nimesulide, rofecoxib, parecoxib, and SC-58236, which have no effect on seizure, even exacerbate seizure severity and increase mortality in animal models (14, 138–141). These contradictory findings in different studies suggest the complexity of neuroinflammatory signal pathways in epileptogenesis.

### 5.9 Mammalian/mechanistic target of rapamycin signaling

The mTOR signaling plays acritical roles in neurodevelopment and neural circuit formation by regulation the protein synthesis and autophagy (18). Recently, several lines of evidences support that mTOR signaling is implicated in neuroinflammation via regulating the function of immune cells and mediating the releasing of pro-inflammatory cytokines and chemokines (142, 143). mTOR signaling may promote the generating of monocytes and macrophages in marrow cavity and promote the transformation of monocytes into macrophages by downregulating the expression of macrophage colony-stimulating factor receptor CD115 (144, 145). mTOR signaling also regulates differentiating and activating of multiple lymphocytes, including T cells and Th cells (18, 146–148). In brain, blocking of mTOR signaling may decrease the formation of autophagosome, increase lipopolysaccharides (LPS)-induced pro-inflammatory cytokines in microglia, attenuate the microglial activation, mitigate astrocyte migration and proliferation, and alleviate seizure severity (18). On the contrary, the activation of mTOR signaling may result in microglial activation and BBB disruption, which facilitate the infiltrating of peripheral immune cells into CNS (149, 150). It is also reported that mTOR signaling regulates the differentiation of Th17 cells and mediates the expression of IL-1β, IL-17 and TNF-α, all of which have been identified to be involved in the epileptogenesis (151–155). Recently, evidences from both lab investigations and clinical trials show treatments targeting mTOR signaling present promising anti-seizure effects, but usually associate with serious adverse events (156).

### 5.10 TLRs

TLRs (TLR 1, 2, 3), a kind of transmembrane proteins expressed by immune cells like microglia and astrocytes, take in important role in recognizing conserved motifs of pathogens or endogenous dangerous molecules secreted by pathological cells. The activating of TLRs subsequently provokes a cascade of innate and adaptive immune response, followed by neuronal hyperexcitability and epileptogenesis (157, 158). The activating of TLRs can be largely enhanced by HMGB1 (159).

Due to the key role on neuroinflammation and regulation of neuronal excitability, TLRs may be potential therapeutic targets in epileptic treatment, which is be reported by several preclinical studies. In a pilocarpine-induced SE model, deletion of TLR3 reduces spontaneous recurrent seizures, inhibits the microglial activation, decreases the levels of pro-inflammatory factors including TNF-α and interferon-β (IFN-β) (160). It is also reported that FOX3P, expressed by microglia and taking part in T cell differentiation, attenuates seizure activity by inhibiting TLR4 signaling pathway (161).

### 5.11 Nucleotide-binding domain-like receptor family pyrin domain containing 3 inflammasome

The NLRP3 inflammasome is an inflammatory complex composed of three proteins, such as NLRP3, apoptosis associated speck like protein containing a CARD (ASC), and cysteinyl aspartate specific proteinase-1 (Caspase-1) (162). By interacting with ASC, NLRP3 inflammasome may induce caspase-1 proteolysis and promote the secretion of a wide spectrum of pro-inflammatory molecules following brain injury in various cerebral diseases (15, 163). In the epileptic brain, NLRP3 is observed to primarily increased in neurons and microglia (164). A growing body of researches show the activating of NLRP3 inflammasome may be involved in epileptic neuron loss, seizures progression, and the therapeutic effects of antiseizure drugs (ASDs) by regulating innate immunity and neuroinflammation (165–169). In rats with amygdala kindling SE, inhibition of the NLRP3 inflammasome downregulates the levels of IL-1β and IL-18, alleviates the severity of epileptic seizures, and decreases hippocampal neuronal apoptosis during the chronic epileptic phase (170). It is also reported that GPR120 downregulates neuroinflammation and alleviates epileptic seizure activity via NLRP3/Caspase-1/IL-1β signals in KA-induced temporal lobe epilepsy (171).

### 5.12 Conclusion

The neuroinflammation, evoked by pathogens, self-antigens, or tissue injury in various cerebral diseases, involves the activation of microglia and astrocytes, a cascade of inflammatory mediator releasing, and infiltration of peripheral immune cells and serum albumin from blood into brain, which subsequently induce the imbalance between glutamatergic signaling and GABAergic signaling, lower seizure threshold and contribute to epileptogenesis (6, 172). Moreover, frequent and continual epileptic seizures also have undesirable consequences on neuroinflammation, thus deteriorating the frequency and severity of epilepsy. During the past two decades, plenty of evidences depending on the animal model or epileptic patients have been demonstrated that neuroinflammation is intricate with the occurrence and development of epileptic seizures.

Although above several established and novel mechanisms are reported to explore the effects of inflammation on neuronal hyperexcitability and the occurrence of epileptic seizure, the current researches predominantly focused on the co-occurrence of both neuroinflammation and epileptic seizures, but could not give a complete insight into the cause-effect relationship between inflammatory molecules and epileptic seizures. The exact role and mechanism of neuroinflammation in epilepsy are far from illustrating. Thus, more in-depth studies are required and indispensable to elucidate the inflammatory mechanisms in epileptogenesis. In future, extensive efforts should be made to further explore the potential crosstalk action between different cells such as activated microglia, astrocytes, peripheral immune cells, and neurons in neuroinflammation, as well as the key crosstalk role of pro-inflammatory factors such as cytokines, chemokines, bioactive lipids, growth factors, etc. In addition, the elevated inflammatory mediators in serum and CSF associated with the seizure severity and recurrence may potentially be explored as biomarkers of refractory epilepsy or epileptogenesis.

Base on the crucial role of inflammation in epileptogenesis, immune modulation and anti-inflammation are excepted to be effective therapeutics for epilepsy. To date, substantial number of preclinical studies have been done and showed that combating neuroinflammation to blocking the development and progression of epilepsy may be an attractive therapeutic strategy for the management of epilepsy. Recently, a wide range of ASDs are available in hospital, but it can’t be ignored the fact that the current ASDs are mainly block the occurrence of seizures but do not act on the underlying pathophysiologic mechanism and epilepsy development (modifying the epileptic process). Different from ASDs, treatment with anti-inflammatory agents may obtain more beneficial effects from neuroprotection to functional recovery in patients with epilepsy, as well as improving the pharmaco-sensitivity to ASDs, but which need further clinical investigations to be done. Therefore, future translational researches should be more focus on evaluating the combined treatments including anti-neuroinflammatory agents and ASDs in patients with epilepsy.

## Author contributions

WL: Writing – original draft. JW: Writing – original draft. YZ: Writing – original draft. WZ: Writing – review & editing.
